# “The Terror of Death Began To Stalk Him”: The Mysterious Fistula of Charles the Wise

**DOI:** 10.7759/cureus.32535

**Published:** 2022-12-14

**Authors:** Matthew D Turner

**Affiliations:** 1 Hospital-Based Medicine, Madigan Army Medical Center, Lakewood, USA

**Keywords:** arsenic toxicity, poison, hidradenitis suppurative, history of medical sciences, medieval, france, charles v, charles the bad

## Abstract

For 23 years, Charles V “The Wise” of France suffered from a mysterious fistula on his left arm that continuously drained pus. For all this time, he believed that as soon as the fistula ceased to weep, he would have a mere 15 days before death followed. His death in 1380, at the young age of 42, seemingly proved this assumption correct. This paper explores the possible explanations from arsenic poisoning at the hands of his longtime nemesis Charles II, as many of his contemporaries believed, to an undiagnosed case of hidradenitis suppurativa, to an underlying tuberculosis infection, to the possibility that his condition was entirely self-inflicted. While it is impossible to determine a definitive cause, it is highly unlikely that Charles II "The Bad" of Navarre had anything to do with his rival's strange condition.

## Introduction and background

For nearly a quarter of a century, Charles V “The Wise” of France suffered from a mysterious illness: a fistula on his left arm that continuously drained pus. A foreboding prophecy had warned the king that as soon as the fistula ceased to weep, he would have a mere 15 days before death followed. His death in 1380, at the young age of 42, seemingly proved this assumption correct. Many of his contemporaries suspected the hand of his longtime nemesis, Charles II “The Bad” of Navarre, in the king’s strange condition. The evidence available to us today does suggest that perhaps Charles II may have poisoned his rival with arsenic around the time that the strange fistula developed. But would this have been enough to cause 23 years of pus drainage? Or was Charles V’s affliction due to another disease process entirely? The key to unraveling this mystery may lay in the decades-long rivalry between the two kings.

## Review

The bad and the wise

 In her book *A Distant Mirror: The Calamitous 14th Century*, the historian Barbara Tuchman describes Charles II of Navarre as a “…small slight youth with glistening eyes and a voluble flow of words… he was a plotter, subtle, bold, absolutely without scruple, but so swerving and unfixed of purpose as to undo his own plots. His only constancy was hate” [1]. In spite of this, even his greatest foes could not deny the “magnetic personality” he possessed, with a “charisma that impressed enemies as well as friends” [2]. The ruler of the Kingdom of Navarre, a small mountain realm located along the modern border between France and Spain, he may have been little more than a historical footnote if not for his ample possessions in Normandy, possessions that, as the Hundred Year’s War between France and England reached its zenith, made him both a crucial vassal to King Jean II of France and an important player in court politics [1]. He spent most of the early years of his reign in France, an ever-present thorn in the Crown’s side [2].

Charles II first emerged onto the political scene at the age of 21 years, in an episode that encapsulated his lifelong approach to the power struggles of the nobility. King Jean II of France had recently granted the county of Angouleme, a holding that Charles regarded as his birthright, to Charles d’Espagne, a royal favorite and the Constable of France. Although Jean II attempted to repair the insult by giving the hand of his daughter, Jeanne, to Charles II, he withheld the dowry for the princess’s hand, only further inflaming the situation. Furious for revenge against his new father-in-law, Charles II had the Constable of France brutally assassinated. On January 8, 1354, his younger brother Phillip led a group of noblemen that broke into the Constable’s room and hacked him to death; by some accounts inflicting 80 wounds upon the dead man’s body before they left [1]. The blatant murder of such an important figure, for reasons, that to most contemporaries, appeared to be motivated by passionate revenge, was only the start of Charles the Bad’s political career.

And yet, thanks in a large part to his eloquence, with which he varnished over his actions his entire life [3], and by threatening to ally with the English crown and provide them a base for the invasion of France, Charles II was soon able to blackmail Jean II into a royal pardon. In a public reconciliation ceremony in Paris, the two in-laws officially forgave one another [1]. Scarcely a year later, Charles II betrayed his liege once again, reaching out to the English and promising to support them in a campaign against the French [1].

It was in this setting that the two Charles (Charles II and Charles V), one a king and one a prince, would first become friends. Thanks to Charles II’s marriage into the Valois royal family, the two young men doubtless knew of one another, but their first significant interactions appear to have occurred in September 1355, when Charles II spent several days at Prince Charles’ (otherwise known as the Dauphin at this time, reflecting his status as heir apparent to the French throne) castle in Vaudreuil. Charles II’s magnetic personality quickly won over the Dauphin [2]. Perhaps further wishing to hurt his father-in-law, Charles II and his co-conspirators quickly set about poisoning the Dauphin against the King. By December 1355, they had concocted a plan to overthrow Jean II. While it is unclear how aware the Dauphin was of this plan, Sumption believed that the young prince had been persuaded that his father would never grant him any degree of true power while he lived, perhaps even that his life was at risk in his father’s court [2]. Jean II soon became aware of the conspiracy and moved to head it off by granting his son the important Duchy of Normandy and paying off much of his financial debts [2].

This was a miscalculation on the king’s part. With the Dauphin acting as the new Duke of Normandy, Charles II had even easier access to the young prince than before. As whispers of conspiracies between the two Charles again spread, by the spring of 1356 Jean II had had enough [2]. In full armor [2], Jean II personally stormed a banquet where the king of Navarre was entertaining the Dauphin. With screams of “Traitor!”, the French king seized Charles II and a handful of his co-conspirators. Several of his allies were beheaded, while the king of Navarre was thrown into prison, his plans dashed [1].

As luck would have it, barely five months later, Jean II found himself a captive as well. The defeat that the French suffered against the English at the Battle of Poitiers on September 19, 1356, was an utterly crushing one; Jean II and a significant chunk of the French nobility were captured and taken to the Tower of London to serve as hostages until the already-bankrupt kingdom could raise the eye-watering ransom that the English demanded [1]. At this point, Charles II may have hoped that his brother-in-law and former fellow conspirator, the Dauphin, now acting as the regent for France, would have freed him. Unfortunately, that did not happen, even as Charles II’s political allies put tremendous pressure on the regent. Perhaps this is what poisoned their relationship, or perhaps it was inevitable that they would become adversaries.

But the cunning king of Navarre had more tools at his disposal than political lobbying. While the French signed a humiliating truce after the battle of Poitiers, the frenzied bloodshed of the Hundred Year’s War did not stop. The English armies may have withdrawn, but massive Free Companies (bands of mercenaries that offered their services to whichever side gave the most coin) still roamed the countryside, burning and looting as they had before. Even from his imprisonment, many believed that Charles II, acting through his brother Phillip, had a hand in funding many of the worst offenders, likely to apply further pressure for his release [1]. Even then, he only managed to escape in November 1357, when a political ally broke into his prison with grappling irons, ladders, and a band of 30 men [2].

Whatever affection or cooperation the brothers-in-law may have displayed in the past was forever gone upon Charles II’s escape. His objectives shifted, and whether out of cold ambition or blazing hatred for the man who had kept him imprisoned for over a year, a wave of violence was unleashed as he made a bid for the French throne. In the maddened political atmosphere, one of his key allies stormed the royal palace at the head of the angry mob, and in front of the Dauphin’s very own eyes, in the very bedchamber of the Prince Regent, had the Dauphin’s two marshals (key political allies) murdered. The attack, so similar to the death of Charles d’Espagne just a few years earlier, was widely believed to have been directly orchestrated by Charles II [1]. Soon afterward, Charles II came within a hairsbreadth of seizing the seat of government but was driven back by the citizens of Paris. As the Dauphin re-secured control of the kingdom, Charles II again forged an alliance with the English and unleashed his forces (Navarrese soldiers, English men-at-arms, and the ever-present Free Companies) to lead a campaign of rapine and slaughter across northern France [1].

 And yet, within a year, Charles II abandoned the campaign against the Dauphin. He revoked his alliance with the English in August 1359 and performed a ceremony of reconciliation with his brother-in-law, just as he had with his father-in-law [1]. Like every agreement he ever made, it wouldn’t last long. By December he was planning a fresh coup that would see an armed overthrow of the regent. Fortunately for the Dauphin, the plan was leaked and never acted upon [1]. Forced to vengefully prowl on the political fringe, Charles II may have turned to more direct measures to eliminate his former ally.

The poisoned prince?

It was around this period that the Dauphin’s contemporaries started to comment on his health. According to the chronicler Froissart, he was “…seized with an illness, which very much disheartened all who loved him; for as no remedy could be found for it, they foresaw that in a very short time he must depart this life… the reports were firmly believed that the King of Navarre, during the time he resided in Normandy, had attempted to poison him…” [4]. The future King of France was wracked with a strange “venom” that caused “the hairs of his head and the nails of his hands and feet (to) fall off...” [4]. Tuchman notes that Froissart may have been correct; the description of Dauphin’s symptoms is remarkably similar to arsenic poisoning [1].

After being treated by a physician named George of Prague, who performed the “…greatest cure known…” by draining out the venom “through a small fistula on (the Dauphin’s) arm” the future King’s symptoms were soon completely resolved [4]. However, in a perplexing turn, the physician warned that the fistula left behind would weep for the rest of his life; the day it stopped draining pus, Charles V would have only 15 days until his death. For the next 23 years, the mysterious fistula drained pus from the King’s left arm, even as his other symptoms resolved [1]. According to Froissart, “…when the fistula started drying up and ceased to weep, the terror of death began to stalk him…” [4]. Just as the physician had predicted, the now-King Charles V died soon after his fistula ceased to drain. He was only 42 years old.

Immediate suspicion fell upon Charles II, at this time an exile in the Kingdom of Navarre. It was widely believed by contemporaries that “… he had caused poison to be administered (when Charles V was) Dauphin; the effects of which, tho’ retarded, or mitigated by medicine, are nevertheless said to have yet eventually terminated in his premature death” [3].

Charles II was no stranger to direct, immediate violence. In addition to his penchant for orchestrating bloody assassinations, his forces slaughtered an estimated 3000 peasants during the ill-fated Jacquerie Rebellion, a 10th of whom were consumed by the flames of a burning monastery [1]. In one particularly gruesome episode from the failed rebellion, Charles II captured one Guillame Cale, the leader of the Jacquerie movement, beneath a flag of truce. The king of Navarre mockingly crowned the peasant “King of the Jacques” with a circlet of red-hot iron before having him beheaded [1]. But poison was another weapon that the king of Navarre knew well. He was noted to have once poisoned one of his Free Company commanders, a bandit named Seguin de Badefol, when the mercenary unwisely asked for his payment [1]. A well-known story that circulated throughout France during his time was that of the tragic death of Gaston, son of the Count of Foix. In it, Charles II’s sister Agnes was said to have come to him complaining of troubles with her adulterous husband, the Count of Foix. Perhaps because of his sister’s distress, or perhaps because he had been in a disagreement with his brother-in-law over finances, Charles sent Agne’s son, the fifteen-year-old Gaston, back to Foix with a bag of mysterious powder, stating that it would help the couple reconcile, but only if the youth added it to the Count’s food in secret. Luckily for the Count, the powder was discovered, and when he fed it to one of his dogs, the animal died in hideous agony. Furious that his son and sole legitimate heir had unwittingly nearly killed him, Foix seized Gaston angrily. At the time the youth had been holding a knife to trim his nails with, and in the confusion, he inadvertently ended up slitting his own throat [1]. As Froissart grimly notes: “It was his father who actually killed him, but the King of Navarre dealt the mortal blow” [4].

It was stories such as this and his vassal’s constant betrayals that made the Dauphin, now King Charles V of France, launch a campaign in 1378 that forever tore Normandy out of Charles II’s grasp. For the rest of his life, Charles was forced to live in the cramped confines of his mountain kingdom, all political influence stripped from him [1]. But not even this setback could deter the king of Navarre’s love of poisonings. A mere two years before Charles V’s death, Charles II’s personal physician, a man named Ángel de Costafort, and a number of other members of the king’s retinue were arrested for plotting to poison the king of France on behalf of their master [5]. It appears that the possibility of being poisoned by his old rival was something Charles V was well aware of.

But the question remains: is it possible that Charles V’s fistula could have been due to poisoning by his lifelong rival?

The possibility

At the time, arsenic was not a difficult substance to obtain and could be found almost ubiquitously in most apothecaries. During the Middle Ages and Renaissance, it was often referred to as the “king of poisons” and the “poison of kings” due to its odorless and tasteless nature [6]. Even the acute symptoms, largely gastrointestinal in nature, can be easily confused with anything from food poisoning to appendicitis [7]. The Dauphin’s symptoms in 1360, hair and fingernail loss, are consistent with arsenic poisoning [7], but the mysterious fistula that lingered for two decades longer remains a pressing question.

While arsenic does not directly interact with DNA, it is co-mutagenic, enhancing the mutating effects of ultraviolet light, and inhibiting the process of DNA repair. This often manifests as hyperkeratosis, altered skin pigmentation, and dermatological cancers [7]. The most common of these are basal cell carcinoma, squamous cell carcinoma, and Bowen’s Disease [8].

Of these, if we assume that Charles V’s weeping fistula was due to chronic exposure to arsenic, the most likely explanation appears to be Bowen’s Disease. This is an in-situ squamous cell carcinoma that typically manifests as a solitary lesion, often with a scaled surface. It is both a persistent and progressive pathology, with approximately a 3% chance of progression to invasive carcinoma [9]. The indolent nature of this disease appears consistent with the skin condition that Charles V suffered from for 23 years.

However, Bowen’s Disease, while attributable to arsenic, is not one that satisfactorily matches Froissart’s description of the ever-draining fistula. The King’s discomfort and constant production of pus are far more consistent with hidradenitis suppurativa (HS). HS is a chronic, recurrent auto-inflammatory skin disease, often found in the axillae or groin, that may be associated with the formation of painful abscesses, deep nodules, and sinus tracts [10]. While it is rare for it to manifest on the extremities, Froissart is not precise in his description of precisely where the mysterious condition was on the king’s arm; it may have been in a more axillary location. While a host of factors go into its etiology, arsenic-induced suppression of the immune system’s signaling mechanisms appears to be pro-inflammatory, suggesting that arsenic exposure may play a role in this disease process after all [11]. The age of disease onset, approximately 23.5 years for men, is also largely consistent with the age of onset of Charles V’s symptoms. While men are less likely to develop the disease in general, they are more likely to develop a severe case, consistent with Charles V’s decades-long disease course [12]. Compared with the unaffected cohorts, those with HS are approximately 50% more likely to develop subsequent cancers [13], possibly contributing to Charles V’s early death.

Rejecting the poison hypothesis

However, while HS is an autoinflammatory condition, the evidence that we present for it being tied to possible arsenic exposure is relatively scarce [11]. If indeed Charles V was exposed to arsenic for a number of months, it would still likely be one factor among many, and it is hardly a convincing argument to suggest that the Dauphin’s possible HS was solely due to the machinations of his rival.

Charles II’s characteristics further support this. While the king of Navarre was certainly capable of intricate plotting, he routinely suffered from a lack of focus [1]. His true agenda is impossible to determine in the modern age, but it appears that he was highly motivated by perceived slights and affronts, and would vengefully lash out in as quick and bloody a manner as possible, as seen by the deaths of Charles d’Espagne, Guillaume Cale, the Dauphin’s two Marshals, and his own nephew, just to provide a handful of examples. In summation, he was dangerous in the short term but unfocused and relatively ineffective in long-time planning.

Even his poisonings were, so far as we now know, acute and fast-acting ones. The dog that ate the poison intended for the Count of Foix died within hours [4]. The mercenary Seguin de Badefol lingered for a mere (and by some accounts, agonizing) six days after eating a crystalized pear laced with poison [5]. While Charles II did have extended access to the future Charles V when the king was still Dauphin, both due to his marriage into the royal family and the Dauphin’s status as Duke of Normandy, it would seem that even had he succeeded in poisoning the prince with arsenic, he was unable to sustain it for a long enough period to kill his future liege. Charles V’s symptoms eventually resolved, and his long-time fistula, whether due to Bowen’s Disease or HS, while possibly worsened by arsenic exposure, cannot be solely attributed to it.

Other possibilities

With both Bowen’s Disease and HS deemed unlikely culprits, a new possibility for Charles’ condition arises, one that is far more consistent with the descriptions of his disease that we currently have available. As Froissart writes, the king’s mysterious fistula developed after George of Prague allegedly drained out the poison from Charles’ arm [4]. Brodie’s abscess, a sub-acute form of osteomyelitis first described in 1832, often presents as a collection of pus in bone, typically without a widespread inflammatory response in the rest of the body. It is far more common in men, often develops at a young age, and may present with swelling, pain, and drainage sinus tracts. Often associated with *Staphylococcus aureus* infection, it may be associated with minor traumas of the bone without an associated fracture [14]. There is also evidence to suggest that Brodie’s abscess may be associated with conditions such as superficial granulomatous pyoderma (SGP), highly associated with chronic, pus-discharging ulcers, with spontaneous healing being infrequent [15]. This raises the intriguing possibility that, in draining the Dauphin’s arm, George of Prague may have inadvertently set the stage for his liege to develop a painful infection.

Even if the king did not have Brodie’s abscess, there are a host of other pathologies that may be responsible for his condition. Tuchman theorizes that he may have suffered from tuberculosis [1]. In the modern United States, of the approximately 20,000 yearly cases of tuberculosis, typically only 11% present as extra-pulmonary [16]; however, this statistic is hardly comparable to the 14th century, which had vastly higher rates of infection [17]. Cutaneous tuberculosis, specifically scrofuloderma, is certainly possible. Often presenting as chronic lymphadenitis, it can also sometimes present as a soft tissue infection that forms sinus tracts with drainage [18]. Interestingly, this pathology, called the “King’s Evil”, was well-known to the medieval world, and it was widely believed that the kings of England and France could cure it with the “King’s Touch”, a “ceremonial laying on of hands believed to cure the lesions, and further, to cleanse the nation of collective transgressions against God” [17]. The practice of the King’s Touch began some 400 years before Charles V and concluded with Charles X of France in 1825 [17]; it seems impossible that this practice would have been unknown to him. The thought of the king suffering from scrofula himself would have been entirely anathema to the medieval mind, and it is interesting in the texts that we have that this potential contradiction never arises either the king did not suffer from scrofuloderma as his contemporaries recognized it, or any thoughts on this matter were silenced. After all, the king’s ability to cure scrofuloderma through his miraculous touch was seen as evidence that his reign was sanctioned by God [17].

However, a cutaneous infection of tuberculosis is not strictly necessary to mimic the king’s symptoms; tuberculosis osteomyelitis may present with swelling, pain, and a bone-to-skin fistula that drains purulent fluid. One case report of an Italian man with right shoulder pain secondary to tuberculosis osteomyelitis [16] reads remarkably like Froissart’s description of Charles V over 600 years earlier. It is almost certain that the king was exposed to the disease at some point, for tuberculosis was incredibly widespread in the medieval world. In an excavation of the late medieval Bácsalmás-Óalmás archaeological site in southern Hungary, researchers found evidence of morphological changes from tuberculosis in at least half of the recovered skeletons [19]. Tuberculosis was so common that King Charles II of England granted over 100,000 of his citizens the “Royal Touch” to cure them of scrofuloderma [17]. It is almost certain that Charles V was exposed to tuberculosis at some point in his life; whether this infected him with a cutaneous scrofuloderma or an osteomyelitic condition is impossible to determine at this time.

There is another possibility as well, one grimmer than Brodie’s abscess or some other form of infection. For 23 years, Charles V emphatically believed that he would die when the fistula ceased to drain [1]. Given this information, it seems logical that the king would do everything in his power to ensure that the fistula continued to drain, perhaps by artificially worsening the condition through mechanical stress, something possible in HS [14]. Another alarming possibility that arises from this line of thinking is that the king’s fistula may have never been due to an underlying dermatological pathology at all; it may have been a completely self-inflicted condition unwittingly caused by George of Prague’s mysterious warning. The “pale, thin, and grave” king who always “lived under a sense of urgency” [1] may have been the author of his own pain.

As for Charles the Bad, the “withered old serpent”, as Tuchman describes him, he lived on for another seven years in exile. After one last plot to poison two of Charles V’s surviving brothers, he finally met his fate in a freak accident that saw him burned alive and left to suffer for two agonizing weeks before he passed in 1387 at the age of 54. It was a fate that his contemporaries felt was well-deserved.

## Conclusions

It appears possible that Charles II may have successfully poisoned the future Charles V, likely with arsenic, around the year 1360. Such an act was consistent with both his previous and future behavior. However, even if this was the case, the Dauphin soon recovered. The strange fistula that he suffered from for the next quarter-century likely had nothing to do with any attempted poisoning. Possibilities range from the unlikely, such as HS, to the likely, such as an undiagnosed Brodie’s abscess, an underlying osteomyelitis, or a tuberculosis infection. It is also possible that the ever-weeping fistula was maintained by the king himself in an attempt to stave off George of Prague’s dire warning.
